# The DREAM Programme, lessons learnt from 20 years of experience

**DOI:** 10.3389/fpubh.2025.1674241

**Published:** 2025-11-20

**Authors:** Anna Maria Doro Altan, Paola Germano, Julien Neze-Sebakunzi, Cristina Cannelli, Flavio Ismael, Fausto Ciccacci, Fatoumata Sylla, Gabriella Bortolot, Maria Cristina Marazzi

**Affiliations:** 1 Department of Life Science, Health, and Health Professions, Link Campus University, Rome, Italy; 2 DREAM Programme, Community of Sant'Egidio, Rome, Italy; 3 DREAM Programme, Community of Sant'Egidio, Kinshasa, Democratic Republic of Congo; 4 DREAM Programme, Community of Sant'Egidio, Maputo, Mozambique; 5 Department of Biomedicine and Prevention, University of Rome “Tor Vergata”, Rome, Italy; 6 DREAM Programme, Community of Sant'Egidio, Conakry, Guinea; 7 DREAM Programme, Community of Sant'Egidio, Genova, Italy

**Keywords:** HIV, Africa, adherence - compliance - persistence, Universal Access to Health Care Services, non-communicable chronic diseases

## Abstract

We describe characteristics and results from the DREAM Program, a public health program initiated in Mozambique in 2002 to fight AIDS and other chronic diseases in Sub Saharan Africa. The DREAM Program is currently implemented in 10 countries providing free-of-charges services to over 110,000 patients. DREAM achieved remarkable success in the prevention of mother to child transmission of HIV, in adherence and retention support, in viral load monitoring. Among the challenges: the limited therapeutic options for people living with HIV experiencing virological failure and limited access to resistance tests.

## Introduction

Diseases Relief trough Excellence and Advanced Means (DREAM) Program is a public health program launched in Mozambique in 2002 as a response by the Community of Sant'Egidio to the injustice of the double standard in the fight against HIV. In fact, an effective therapy had existed since 1996 but it was not widespread in Africa. One of the reasons was the resistance and skepticism of the international community in addressing a problem of such dimensions in contexts with few resources (1, 2). However, Sub Saharan Africa was, and still is, by far, the region most affected by the disease, as emerged at the 2000 Durban conference (3), which represented a real turning point. A response was required both for humanitarian reasons and for global security and stability. The first years of the DREAM program were therefore dedicated to bringing therapy to countries where it did not exist (4), and to demonstrating the concrete feasibility of the treatment (5). The momentum for change happened between 2001 and 2003 (6), with the announcement and funding of Global Fund, and the creation of PEPFAR. Consistent and growing financing allowed the real scaling up of antiretroviral treatment in Sub Saharan Africa. The treatment and prevention of this contagious and incurable disease in sub-Saharan Africa has been and continues to be a challenge of immense proportions, given also the high number of people living with HIV (PLWH) in the continent. Among the problems to be addressed are: the fragility and chronic under financing of health systems, with shortage of human resources, insufficient investment in health professionals education, insufficient infrastructures and lack of diagnostic equipment; the stigmatization linked to AIDS, due to the sexual way of transmission but also to the inevitably fatal course of the disease; high cost of drugs; lack of awareness of the population in approaching chronic diseases; the high number of women infected, if compared with other regions, and consequently the high number of infected children.

DREAM Program tried to respond to these challenges in an innovative way, combating the idea of therapeutic and diagnostic minimalism and proposing high quality therapeutic and diagnostic protocols, more similar to those applied in high income countries.

## Context

**Figure 1:**
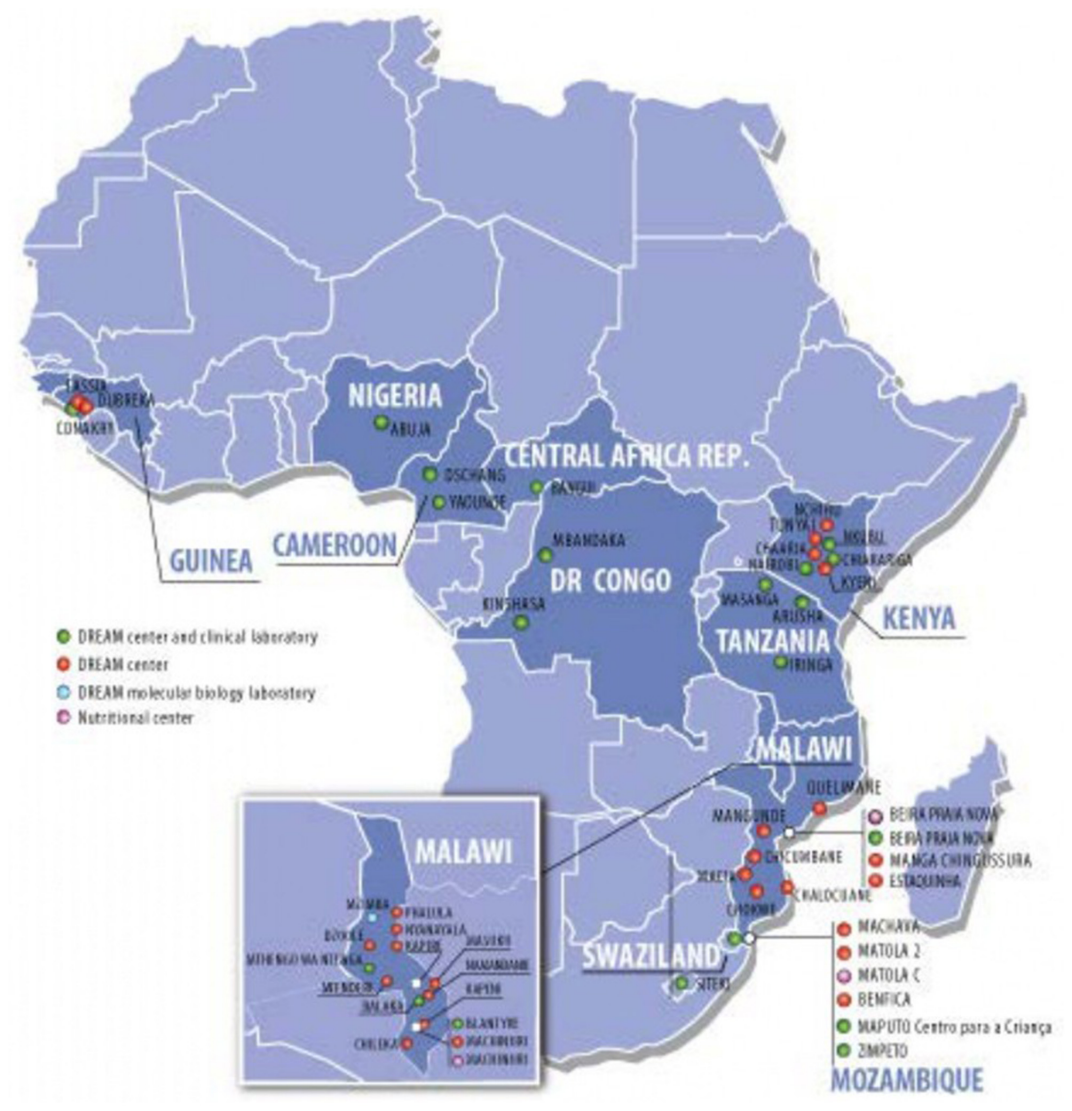
Geographical distribution of DREAM Centers and laboratories.

The DREAM Program is currently implemented in 10 countries in Sub Saharan Africa (Figure 1): Malawi, Mozambique, Tanzania, Guinea, Democratic Republic of Congo, Cameroon, Eswatini, Kenya, Central African Republic, and Nigeria. It offers a wide range of services: HIV diagnosis and treatment, prevention of mother to child transmission of HIV, tuberculosis diagnosis and treatment, hypertension screening and treatment, nutritional interventions, screening and treatment of epilepsy and other neurological conditions, screening and treatment of precancerous lesions of the cervix, vaccinations; its 50 outpatient clinics and 28 molecular biology laboratories are integrated into national programs to fight HIV and tuberculosis, working in collaboration with the Ministries of Health of the respective countries. The above mentioned facilities offer free-of-charge treatment and care directly to more than 90,000 people living with HIV and 20,000 patients suffering from other chronic conditions, and indirectly contribute to the care of other 400,000 PLWH through laboratory services.

## Detail: key programmatic elements

Characteristics of the program have been described elsewhere (6–10). Some key elements are outlined here.

From the beginning, the DREAM program has placed an emphasis on strengthening diagnostic capacity, which has been identified as a bottleneck in Sub Saharan Africa (11). We have developed the molecular biology section and HIV-related diagnostics, alongside other basic analyses.

The choice was made to set up outpatient clinics rather than hospitals, given the difficult sustainability of the latter and the chronic nature of HIV; in fact, a lack of specific services for chronic diseases was identified.

A special attention is paid to reinforcing adherence and retention in care of PLWH and people living with other chronic conditions through a diverse set of interventions: building trusting relationships between the staff and the patients; investing in the therapeutic literacy of patients; support groups; individual counseling carried out by all members of the team; a policy of free-of-charge visits, diagnostic tests, drugs; inclusion of people with HIV in the professional team of the center. The relationship of trust between patients and operators that has been built over the years has allowed us not to lose people even in pandemic crises such as Ebola in 2014 in Guinea or COVID in 2020–21. The DREAM centers have always remained open even in the most difficult moments, and even when they were destroyed, such as during cyclone Idai in Beira, the staff worked to ensure continuity of care.

In the DREAM program, people with HIV play a fundamental role, some of whom have become leaders in the fight against AIDS, transmitting hope to other patients. Others are peer educators, others are an integral part of the care team as coordinators, nurses, doctors. Through them, strong linkage and relations are maintained between the clinic and the local community.

Another key element is the use of information technology: a dedicated software has been created to manage electronic patient files, pharmacy, laboratory results, scheduled appointments. The system is not primarily conceived for collecting data and producing statistics, but to help health staff in managing more efficiently and with fewer errors a consistent workload and in supporting retention in care.

## Achievements and challenges

A first remarkable success of the Programme is the elimination of mother-to-child transmission, which has a clear impact on the health of future generations; the efficacy of antiretroviral therapy, the determination of women in wanting to protect the health of their children, as well as the support activities carried out by expert patients of the programme, allow to obtain excellent results, that is, a transmission rate consistently below 2% (10, 12, 13).

Among the other elements of success of the program, we would like to highlight the good level of achievement of some indicators that according to the WHO define a good quality of the programs, the so called “early warning indicators of HIV drug resistance” (14). Among them are the retention in care at 12 months, viral load testing coverage, viral load suppression.

**Figure 2:**
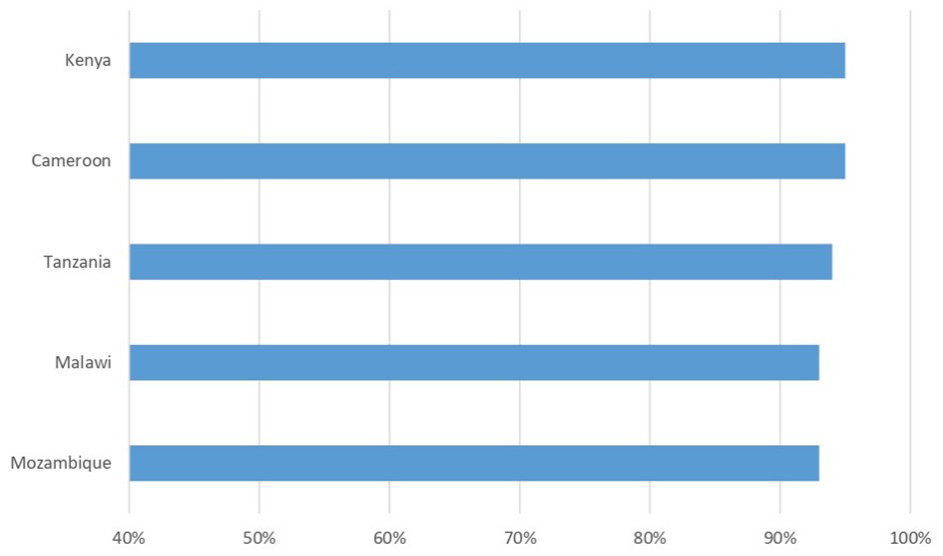
Percentage of patients retained in care after 12 months. Baseline: 65,191 patients followed in DREAM centers at the end of 2022.

Retention in care once treatment has started is an important goal in a chronic disease like HIV, whose therapy is lifelong; people with HIV need to be motivated and supported in the face of normal “therapeutic fatigue,” especially when, as periodically happens, fake news of miraculous healings spread. Figure 2 shows data from a cohort of 65,191 patients followed in DREAM centers in five countries at the end of 2022, and the percentage of them being still in the treatment center at the end of 2023. All patients in care on December 31th 2022 in the five countries were included, they represent the 74% of the 88,257 patients in care in the 10 countries. A patient was considered in treatment in the center if he/she had not an outcome of “death,” “transfer out” or “lost,” this last outcome refers to patients missing a scheduled drug refill appointment for more than 3 months. Retention rates vary from 93 to 95%.

**Figure 3:**
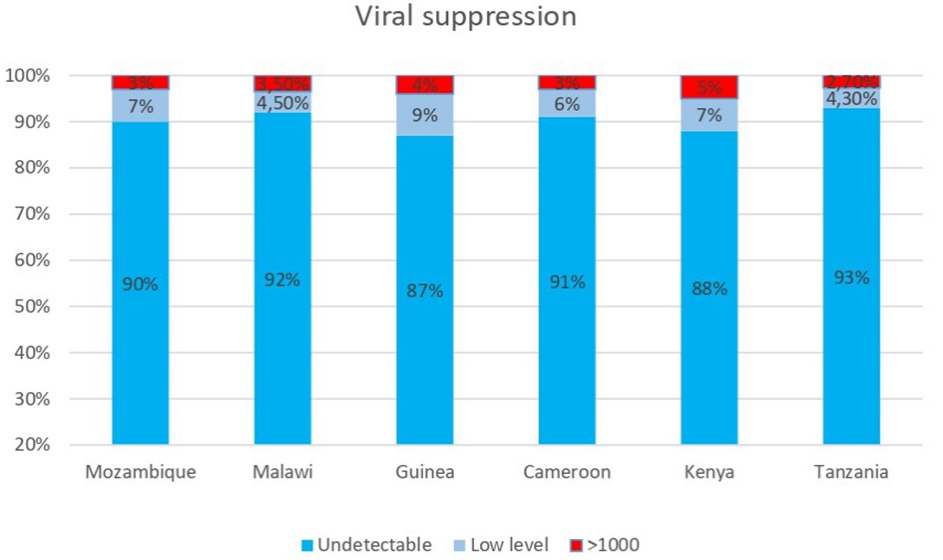
Viral load results from 66,707 patients on ART in DREAM centers in six countries who received a VL measurement in 2023.

Regarding viral load testing coverage, more than 95% of DREAM patients benefit from the free viral load test every year, achieving the target established by WHO: among them, more than 95% have a viral load lower than 1,000 copies; these values have improved after the transition to Dolutegravir-containing regimens for most patients. Figure 3 shows data of viral suppression on a cohort of 66,707 patients who had a viral load measurement in 2023. It is also important to stress that almost 90% have a viral load < 50 copies/ml, achieving this threshold is the best way to prevent the development of drug resistance.

Among the challenges we are facing, we have to mention the limited therapeutic options for the minority of patients who have not achieved virological suppression. One element of concern is that, in a recent study carried out in Mozambique (15), we have seen the emergence of resistance to Dolutegravir (DTG), the antiretroviral drug of recent introduction in Africa (since 2018), which is now used by more than 90% of patients, the drug theoretically most durable, which according to clinical studies (16) promised to create little or no resistance. In our cohort, patients who experienced confirmed virological failure were eligible for testing; it was possible to perform only a small number of tests, due to high costs and complexity of the exam; among the 19 samples tested for DTG resistance, nine had some degree of resistance. Other studies in Africa show similar results (17, 18). Another cause for concern is that the various national guidelines, with a few exceptions, do not provide guidance on what to do in the event of resistance to Dolutegravir and also very few patients in Africa have access to resistance testing.

**Figure 4:**
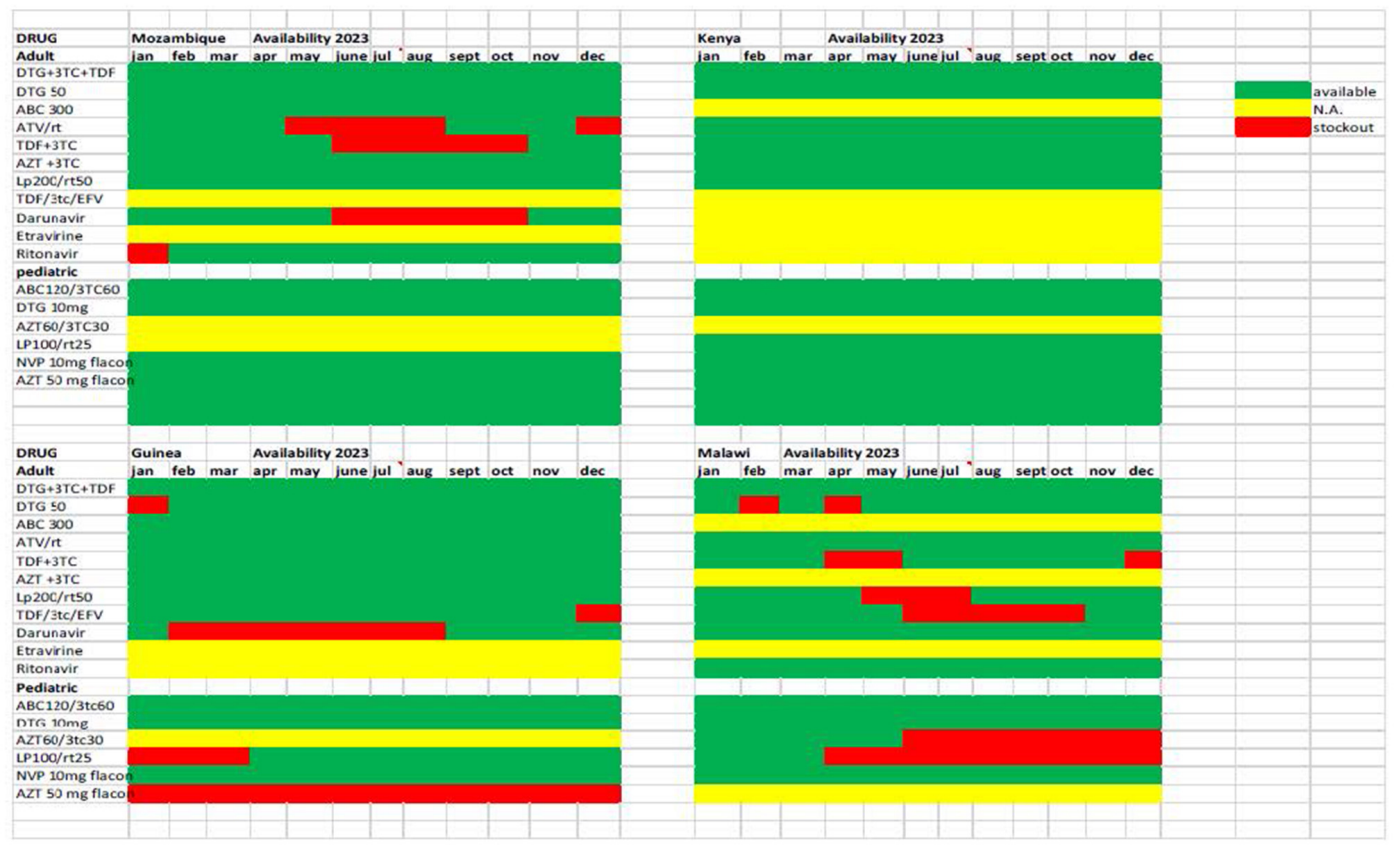
Availability of ARV drugs in pharmacies of DREAM Centers in four countries during calendar year 2023.

A second challenge related to this is the limited availability of different classes of antiretroviral drugs in the real life of health facilities. The DREAM centers are included in the normal national drug supply chain. The graph (Figure 4) shows the availability during 2023 of the combinations of drugs recommended by the WHO guidelines and used in the various countries; these are different combinations of about 10 antiretroviral drugs, compared to about 25 used in Europe and the USA. Over the years an effective supply chain has been established at a national level for the most used combinations, while for other drugs there have been stockouts of months in almost all countries, and some combinations (in yellow) are not available at all. Precisely in light of the possible increase in the number of patients with resistance to Dolutegravir, it would be important to have a wider variety of drug combinations, in order to carry out a personalized therapy.

A further challenge is certainly that of combating and overcoming stigma and in some cases discrimination. Although the life of people with HIV in the vast majority of cases is now an absolutely “normal” life, for many HIV is not a disease like any other. It is in fact a condition that involves many aspects of life, not only physical health but also family relationships and social life. Table 1 shows the results of two surveys, one conducted in Mozambique on HIV-negative people, the other in Guinea on 400 people with HIV, DREAM patients. More than half of the interviewees think that HIV is not a disease like any other, and would not tell anyone that they are HIV positive. When asked “what were your reactions when you found out you had HIV,” 32% had reactions of strong despair, shock, and 9% thought about suicide. But we also see that 35% of the people felt encouraged by the advice and support of the staff and regained hope in living. These data support the practice that has been consolidated over many years of always accompanying the HIV test with a moment of counseling, of dedicating time to this diagnosis that has such an impact on people's lives.

**Table 1:** Results from two surveys on stigma and disease perception.

**Questions and answers by country**	***N* (%)**
**Mozambique (general population)**	**111 (100%)**
Is HIV like any other disease?	
No	61 (55%)
Yes	42 (38%)
I don't know	8 (7%)
If you were seropositive, would you disclose?	
No	55 (50%)
Yes	49 (44%)
I don't know	7 (6%)
**Guinea (PLWH)**	**402 (100%)**
Which were your reactions when you learnt you were HIV positive? (more than 1 answer)	
Cried a lot	140 (35%)
Thought my life was finished, shocked	128 (32%)
Wanted to commit suicide	36 (9%)
Felt encouraged by your counseling and regained hope	141 (35%)

DREAM program dedicates efforts to reach and maintain in care those who have the greatest difficulty in accessing and remaining in health services. We have realized how often men are left behind in accessing care. For cultural and work-related reasons, men attend health services less than women, are tested less frequently, also because one of the main access points to testing is pregnancy, and their mortality rates for HIV are higher than those of women. Our health centers are mostly attended by women, however, the low male involvement in care often also affects the adherence of their partners. Through the “WeMen” (19) study, funded by the Italian Cooperation, we tested three different interventions to increase male involvement in Malawi. Among these, the one that was most satisfying for the participants, and that generated a lasting greater involvement, was that of the “special days,” in which on dedicated days, that is one Saturday a month, a package of health services was offered specifically for men. Therefore, men feel the need for a health intervention adapted to their needs and their schedules.

Another particularly vulnerable and difficult to reach population is that of adolescents with HIV. Some of them were infected at birth, and, as they grow up, they often develop a sense of rebellion toward life, the disease, and refuse to continue treatment; others become infected at the beginning of sexual activity, and, due to gender inequalities, these are much more often girls (20); adolescents and young people have lower retention than adults, they take the HIV test less often, and have lower virological success (21). To meet the special needs of this population we have used various approaches: group meetings, interviews with young counselors; training for all health personnel on adolescence, and on how to communicate with adolescents; the testimony of adults with HIV who are personalities in the professional, sports or musical fields. Through some of these initiatives we obtained a documented increase in retention (22, 23).

## Discussion and lessons learnt

DREAM Programme was born in Mozambique and subsequently spread to other nine countries in Africa. These countries share some similarities, but also significant differences. First of all, DREAM works in countries with a high prevalence of HIV (especially in Southern and Eastern Africa) as well as in countries with a lower prevalence (Central and western Africa). This translates into different patient influx and workload on health centers, and consequent organizational changes. Another important difference is the structure of health systems, in terms of availability and qualifications of the healthcare workforce, which has led in some countries to a task shift toward less specialized resources. Accessibility of basic health services also vary across countries, for this reason DREAM centers adapted in order to offer a wider range of services. Finally, religious, cultural and family law differences translate into different approaches to the disease and varying degrees of stigma. Nevertheless, the DREAM model, with the key programmatic elements described above, has been suitable for these different context, and the results and success are consistent across countries. The greatest differences are found, even in terms of retention, between rural and urban settings.

Despite the success of DREAM program and other HIV programs in eliminating mother to child transmission, unfortunately, in the countries where we work, the transmission rate is actually much higher, up to 10% in Mozambique and 20% in Guinea. Reasons for this failure are probably lack to access to HIV testing and ART in early pregnancy and early drop out of pregnant and lactating women (24). It seems therefore necessary to promote access without barriers (i.e., economic barriers or stigma related barriers), to quality prenatal care, including HIV testing and therapy, to truly achieve the goal of eliminating mother-child transmission.

Reinforcing adherence in vulnerable population is crucial especially for prevention as well as for care of chronic conditions. DREAM Programme model stress the importance of trustful relationship between staff and patients, and of the action of peer to peer educators. These elements have been outlined also in studies investigating strategies to increase vaccine compliance (25–27).

A major change occurred during these 20 years of activity of DREAM Program is that, thanks to the effectiveness of therapy, today the life expectancy of people with HIV is almost the same as that of those who do not have HIV, if therapy is started in time (28). On the one hand, this is a call on action for rapidly reaching the people who are left behind, because today deaths from HIV are avoidable; on the other hand, it has posed the question of also treating all those other diseases that appear in a cohort of people followed for years and that are aging, such as hypertension, diabetes, and various cancers. DREAM is also a program for the treatment of these diseases in primary health services, albeit with the limitation of the cost of drugs that are not covered by any fund. DREAM met the criteria for an effective integrated healthcare delivery of health services for chronic conditions as outlined by a consensus of expert (29), such as improved data collection and surveillance of NCDs among people living with HIV, strengthened drug procurement systems, availability of equipment and access to relevant blood tests, health education for all chronic conditions, and enhanced continuity of care for patients with multimorbidity. In Sub Saharan Africa, an interesting model of integrated care delivery is the South Africa's IDEAL clinic (30), which, similarly to DREAM Programme, builds on the strengths of the HIV program to deliver integrated care to patients with chronic and/or acute diseases.

In our experience, treatment programs set up to combat HIV have leveraged effects on the health of the whole population, by introducing investments that benefit the entire healthcare system, as already observed by the WHO (31, 32).

DREAM Programme has chosen a free of charge approach in order to allow access to a high quality care also for the most vulnerable part of the population, and in order to enhance retention in care. This choice was aimed also at responding to the problem of the catastrophic health spending in Africa. According to WHO, in 31 countries in the African Region, the share of out-of pocket health spending exceeded 25% of their current health spending (33). A 2024 WHO report outlines that high health-care costs in Africa continue to push over 150 million into poverty (34). The HIV global response has been pivotal in promoting universal health coverage in Africa, by defining comprehensive interventions and service delivery packages and by championing health access strategies which have reduced the price of health commodities and improved the efficiency of service delivery. After more than 20 years of activity, DREAM Programme shows the feasibility of rapidly scaling up clinical and public health programmes in challenging environments. We think that this success can be replicated for other health conditions.

At the beginning of the fight against HIV in Africa, oversimplified strategies were proposed that turned out to be short-lived, such as the single tablet of nevirapine for the prevention of mother-to-child transmission, staging of the disease done only on a clinical basis, prescription of ART only in the advanced stages of the disease (35). Progressively, especially since 2010, the treatment protocols have come closer to those of advanced countries. At present, the main difference between protocols for low income countries and those for high income countries is the use and availability of drug resistance testing.

In conclusion, the history of the DREAM program shows how even in countries with limited resources it is possible to implement high-quality care interventions. HIV remain a complex disease and oversimplified interventions are unlikely to yield sustainable results. Finally, the protagonists of the fight against HIV are people: the people living with HIV and people who work to treat and prevent, so investing in people is the key to lasting success.

## Data Availability

The raw data supporting the conclusions of this article will be made available by the authors, without undue reservation.
